# Incinerator Pollution and Child Development in the Taiwan Birth Cohort Study

**DOI:** 10.3390/ijerph10062241

**Published:** 2013-05-31

**Authors:** For-Wey Lung, Tung-Liang Chiang, Shio-Jean Lin, Bih-Ching Shu

**Affiliations:** 1 Taipei City Hospital, Songde Branch, Taipei 110, Taiwan; E-Mail: forwey@seed.net.tw; 2 Department of Psychiatry, National Defense Medical Center, Taipei 114, Taiwan; 3 Institute of Health Policy and Management, College of Public Health, National Taiwan University, Taipei 106, Taiwan; E-Mail: tlchiang@ntu.edu.tw; 4 Department of Pediatrics, National Cheng Kung University, Tainan 701, Taiwan; E-Mail: sjlin@mail.ncku.edu.tw; 5 Institute of Allied Health Sciences and Department of Nursing, National Cheng Kung University, Tainan 701, Taiwan

**Keywords:** incinerator, breastfeeding, Taiwan Birth Cohort Study, TBCS-DI, PCC

## Abstract

This study aimed to investigate the direct and indirect effects of environmental pollutants on child development and parental concerns. It focused on the pathway relationships among the following factors: living within three kilometers of an incinerator, breastfeeding, place of residence, parental concerns about development, and parent-perceived child development. The Taiwan Birth Cohort Study (TBCS) dataset includes randomized community data on 21,248 children at six, 18, and 36 months of age. The Parental Concern Checklist and the Taiwan Birth Cohort Study-Developmental Instrument were used to measure parental concern and parent-perceived child development. Living within three kilometers of an incinerator increased the risk of children showing delayed development in the gross motor domain at six and 36 months. Although breastfeeding is a protective factor against uneven/delayed developmental disability (U/DDD), children living near an incinerator who were breastfed had an increased risk of U/DDD compared with those who did not live near incinerators. The presence of a local incinerator affected parent-perceived child development directly and indirectly through the mediating factor of breastfeeding. Further follow-up of these children to investigate the long-term effects of specific toxins on their development and later diagnostic categorization is necessary.

## 1. Introduction

**Figure 1:**
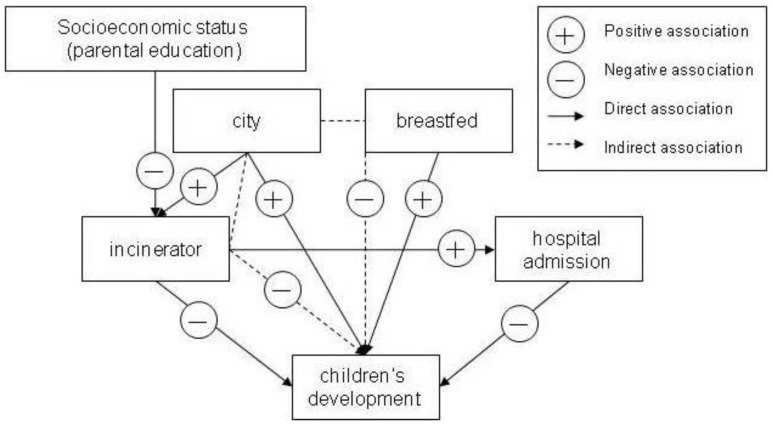
Hypothetical model of the pathway relationships among parental level of education, place of residence, living near an incinerator, breastfeeding, hospital admission, and child development.

The management of municipal and industrial waste is a growing problem throughout the World, and incinerators have been deemed to be a “quick fix” for the problem of waste [1]. However, few studies have investigated the effects of pollutants on children’s motor and psychosocial development. The immature brain of a child is vulnerable to environmental exposure, and the bodies of children are also less effective at detoxifying chemicals than those of adults. As a consequence, the reaction of children to hazardous chemicals differs from that of adults [2]. Most previous studies have focused on the effect of incinerators on children’s physical health [3]. Air pollution is suspected to be related to an array of health problems whose prevalence has increased without explanation [4,5,6,7]. These problems include infertility, autism, attention deficit and hyperactivity disorders, childhood brain cancer, and acute lymphocytic leukemia [4,5,6]. In the case of Metropolitan Mexico City, it was found that children exposed to air that is polluted with urban fine particulate matter experience serious detrimental effects including neuroinflammation, neurodegeneration, and cognitive deficits [7]. Previous studies have investigated the effect of environmental pollution on autism spectrum disorder (ASD); the results showed that environmental factors, including air pollution, organophosphates and heavy metals, have a consistent role in systemic and central nervous system pathophysiology, including oxidative stress, neuroinflammation, and mitochondrial dysfunction [8]. Furthermore, the majority of current evidence points toward an association between mercury and ASD [9,10]. In addition, heavy metals, dioxins, and pesticides can cause neurodevelopmental disorders [11]. Studies of human exposure to dioxins and dioxin-like compounds from waste incineration have shown dioxin levels in blood and milk to be significant biomarkers of exposure [12,13]. Given that human milk can contain traces of dioxins [14], breastfeeding under such circumstances increases the exposure of infants to pollutants [14,15]. Therefore, in addition to direct exposure, children can also be affected by indirect exposure through the oral intake of pollutants [16]. This implies that toxins can be transmitted through breastfeeding by mothers who are exposed to pollutants. Although the adverse effects of pollutants on children have been investigated in previous studies, most have reported the effects on physical health [3,17,18], and few community epidemiological studies have studied the long-term effects of such exposure on child development in Taiwan. Therefore, the purpose of this study was to investigate the direct and indirect effects of living near an incinerator on parental concerns and parents’ perceptions of child development, using randomized community data on 21,248 children at six, 18 and 36 months of age. In addition to living near an incinerator, we also considered important confounding factors that could influence children’s health and development, and the socioeconomic conditions of the parents based on education level. Overall, the study focused on the investigation of the pathway relationships among factors including parental level of education, place of residence, living near an incinerator, breastfeeding, hospital admission, and child development, by using the hypothetical model shown in Figure 1.

## 2. Experimental Section

### 2.1. Participants

The Taiwan Birth Cohort Study (TBCS) is a national household probability sampled study: all babies born between October 2003 and January 2004 in Taiwan were eligible for inclusion in the TBCS, with no exclusion criteria. In order to build a sample that would be representative of the children in Taiwan, a two-stage stratified random sampling method was applied. In the first stage, the primary sampling unit in was cities and towns; 85 were selected from 369 townships in Taiwan by systematic random sampling and then grouped into 12 strata according to four levels of urbanization and three levels of total fertility rate. In the second stage, newborns were proportionally selected, with probability related to the rate of birth, from the 85 selected cities and towns. The final sample comprised 21,248 newborns, with a selection rate of 11.7% at the age of six months. At the 18-month follow-up, 20,172 (94.94%) of these families agreed to remain in the study, and 19,908 (98.69%) remained in the study at 36 months [19].

After the parents had agreed to participate, a trained researcher visited their homes and asked them questions using an interview booklet. The questions in the booklet were designed to collect data on all variables that may affect children’s health and development, including sociodemographic indicators and contexts (child development, family demographics, family process and environmental contexts); location and condition of the home as perceived by the researcher; problems during pregnancy (the delivery method, problems during delivery, and health status of the infant at birth); feeding and infant care conditions; and any issues of adjustment, which targeted spouses from foreign countries in cross-cultural marriages [19].

The TBCS protocol was approved by the institutional review board of a teaching hospital in Taiwan and, after detailed explanation of the study, informed consent was obtained from the parents of all participants at each stage of the study [19].

### 2.2. Materials

All information collected was from parent self-report. At six months, demographic information and environmental data were collected from the main caregivers, including the location of their dwelling (near an incinerator or not) and whether they breastfed their child. Information on the main type of exposure, namely the presence of an incinerator, was collected by asking the parents whether there were incinerators within three kilometers of their place of residence. The health effect was measured by parental reports of their children’s development at six, 18, and 36 months. Information on the developmental condition was collected using the Taiwan Birth Cohort Study-Developmental Instrument (TBCS-DI) [20,21,22], and, at 18 months, data on the concerns of the parents regarding the development of their children were collected using the Parental Concern Checklist (PCC) [22].

#### 2.2.1. Taiwan Birth Cohort Study-Developmental Instrument (TBCS-DI)

The TBCS-DI is a parental-report instrument that measures child development on the basis of parental observation of the child’s daily performance. It evaluates the child’s development with respect to four dimensions: (1) gross motor skills, (2) fine motor skills, (3) language/communication (language), and (4) social/self-care ability (social). There are 26 items in the scale used at six months, 17 in the scale used at 18 months, and 19 at 36 months. The items are evaluated using a three-point Likert scale; higher scores imply better development. The TBCS-DI has shown high reliability, internal consistency, and validity [20,21,22].

#### 2.2.2. Parental Concern Checklist (PCC)

The PCC is a broad problem-oriented screening instrument that is used to identify children with delayed development and is suitable for the Taiwanese culture and language. The PCC is a parent-report checklist consisting of eight items that are used to record parental concerns regarding their child’s development in the gross motor, fine motor, language, and social domains. It was developed from the Parents’ Evaluation of Developmental Status Instrument [23], with modifications to suit the structure of the TBCS-DI [22]. Higher total scores in the PCC imply that parents are more concerned about their child’s development, and cut-off points of 2/3 and 6/7 were used to separate the children into three groups on the basis of developmental level: (1) normal, (2) suspected mild uneven/delayed developmental disability (U/DDD), and (3) moderate U/DDD [21]. The instrument shows good validity and reliability [21]. The U/DDD category includes typical developmental delay, ASD, attention deficit hyperactivity disorder, and other socio-emotional disability disorders that might increase parental concern [21].

### 2.3. Statistical Analysis

The demographic distribution of the infants and parents was analyzed using SPSS 15.0 software for Windows (Chicago, IL, USA). The data for participants who were lost to follow-up at 18 months were replaced using Bayesian analysis, which is an approach that uses all the available information to produce a maximum likelihood estimate. The combined use of Bayesian analysis and pathway analysis to fill in missing data has been found to be ideal for longitudinal studies of child development [19]. In addition, pathway analysis was used to analyze the relationship pathways among the variables of interest. It can also control for bias associated with confounding factors related to the variables of interest [19]. For the pathway analysis, the chi-square distribution was used to test the overall fit of the data. *P* values greater than 0.05, an adjusted goodness-of-fit index (AGFI) greater than 0.09, a root mean square error of approximation (RMSEA) less than 0.08, and a Tucker–Lewis Index (TLI) and Comparative Fit Index (CFI) close to 1.0 show a good fit, which indicates that the model describes the observed data adequately. In addition to the variables of interest (breastfeeding and living near an incinerator), potential confounding factors, including the children’s demographics (gender, twin), children’s health condition (birth weight, gestational age, method of birth, hospitalization, gastrointestinal illness, vaccination), and parental demographics (parental age and level of education) were all controlled in the pathway analysis. The relationships among these investigated variables are represented by beta (β) values of regression or path coefficients. However, the models presented in the results are parsimonious pathway analysis models, which means that only statistically significant pathways (*p* values less than 0.05) are presented. Both the Bayesian analysis and pathway analysis were carried out using the AMOS 7.0 statistical software package (SPSS, Chicago, IL, USA) in November 2011.

## 3. Results and Discussion

### 3.1. Results

#### 3.1.1. Demographic Information

Of the 21,248 children who participated in the study, 953 (4.5%) lived near an incinerator. Approximately half of the participants were male (52.5%), and 2.6% of the children were one of a twin. The demographics of the children and their parents who lived near an incinerator were compared with those of the children and parents who did not (see Table 1).

#### 3.1.2. Parent-Perceived Children’s Developmental Condition and Parental Concern regarding Children’s Development

The results showed statistically significant differences between the two groups with respect to the number of children who were being breastfed at six months and the number who lived in the city (F = 10.86, *p* = 0.026; F = 10.86, *p* = 0.001). The children’s developmental condition at six, 18, and 36 months of age, as measured using the TBCS-DI (gross motor, fine motor, language, and social dimensions), were also compared between the two groups (see Table 2). The results showed that only gross motor development at 36 months was statistically significantly different between the two groups (F = 10.86, *p* = 0.008). The prevalence of both mild and moderate U/DDD, as measured using the PCC, showed significant differences between those who lived near an incinerator and those who did not (χ^2^ = 4.35, *p* = 0.037; χ^2^ = 6.70, *p* = 0.010, respectively), as shown in Table 2.

**Table 1:** Comparison of the demographics of the children and their parents who lived near an incinerator and those who did not (N = 21,248).

		Lives near an incinerator (n = 953)	Does not live near an incinerator (n = 20,295)		
		n (%)	n (%)	χ^2^	*p*
Boys		524 (55.0)	10,621 (52.3)	2.57	0.058
Twins		534 (2.6)	926 (2.6)	0.14	0.391
Breastfed at six months		230 (24.1)	4,348 (21.4)	10.86	0.026
*Children’s health:*					
	Received vaccination	950 (99.7)	20,272 (99.9)	3.02	0.109
	Has had gastrointestinal illness	182 (19.1)	4,054 (20.0)	0.44	0.267
	Has been hospitalized	127 (13.3)	2,658 (13.1)	0.04	0.438
	Lives in the city	503 (52.8)	9,605 (47.3)	10.86	0.001
	Has moved	26 (2.7)	509 (2.5)	0.18	0.375
*Maternal education:*					
	Illiterate	0 (0.0)	18 (0.1)		
	Elementary school	54 (5.7)	774 (3.8)		
	Junior high	103 (10.8)	2,200 (10.8)		
	High school	364 (38.2)	8,134 (40.1)		
	University/college	393 (41.2)	8,467 (41.7)		
	Graduate school	39 (4.1)	702 (3.5)		
*Paternal education:*					
	Illiterate	0 (0.0)	3 (<0.1)		
	Elementary school	16 (1.7)	288 (1.4)		
	Junior high	133 (14.0)	2,503 (12.3)		
	High school	369 (38.7)	8,100 (39.9)		
	University/college	362 (38.0)	7,868 (38.8)		
	Graduate school	1,533 (7.6)	1,533 (7.6)		
**Variable (range)**		**Mean (SD)**	**Mean (SD)**	***t*-test**	***p***
*Parental education (years)*					
	Maternal education (0–18)	12.56 (2.86)	12.68 (2.69)	2.89	0.089
	Paternal education (0–19)	12.92 (2.59)	12.99 (2.51)	1.50	0.221
*Parental age (years)*					
	Mother’s age (14–49)	29.57 (4.91)	29.38 (4.89)	0.09	0.762
	Father’s age (17–80)	33.43 (5.25)	33.30 (5.46)	1.55	0.214

**Table 2:** Comparison of child development, using the Taiwan Birth Cohort Study-Developmental Instrument (TBCS-DI) and the Parental Concern Checklist (PCC), between those who lived near an incinerator and those who did not.

		Lives near an incinerator (n = 953)	Does not live near an incinerator (n = 20,295)		
**TBCS-DI**		**Mean (SD)**	**Mean (SD)**	***t*-test**	***p***
*Six-month development*					
	Gross motor	22.52 (3.38)	22.62 (3.27)	3.12	0.078
	Fine motor	16.24 (1.74)	16.25 (1.73)	0.35	0.553
	Language	20.87 (2.27)	20.96 (2.37)	2.74	0.098
	Social	5.79 (1.36)	5.89 (1.42)	1.99	0.158
*18-month development*					
	Gross motor	13.81 (1.45)	13.88 (1.40)	2.64	0.104
	Fine motor	7.89 (1.23)	7.92 (1.21)	0.27	0.601
	Language	9.91 (2.07)	10.01 (2.12)	0.68	0.410
	Social	13.12 (1.80)	13.16 (1.72)	0.81	0.367
*36-month development*					
	Gross motor	16.63 (1.85)	16.77 (1.70)	6.93	0.008
	Fine motor	10.00 (1.81)	10.03 (1.73)	2.74	0.098
	Language	11.83 (0.86)	11.84 (0.76)	1.75	0.186
	Social	13.78 (1.54)	13.76 (1.49)	0.03	0.864
**PCC**		**n (%)**	**n (%)**	**χ^2^**	***p***
	18-month mild U/DDD	137 (14.4)	2458 (21.2)	4.35	0.037
	18-month moderate U/DDD	20 (2.1)	236 (1.2)	6.70	0.010

#### 3.1.3. Pathway Analysis of Children’s Development at Six, 18, and 36 Months

**Figure 2:**
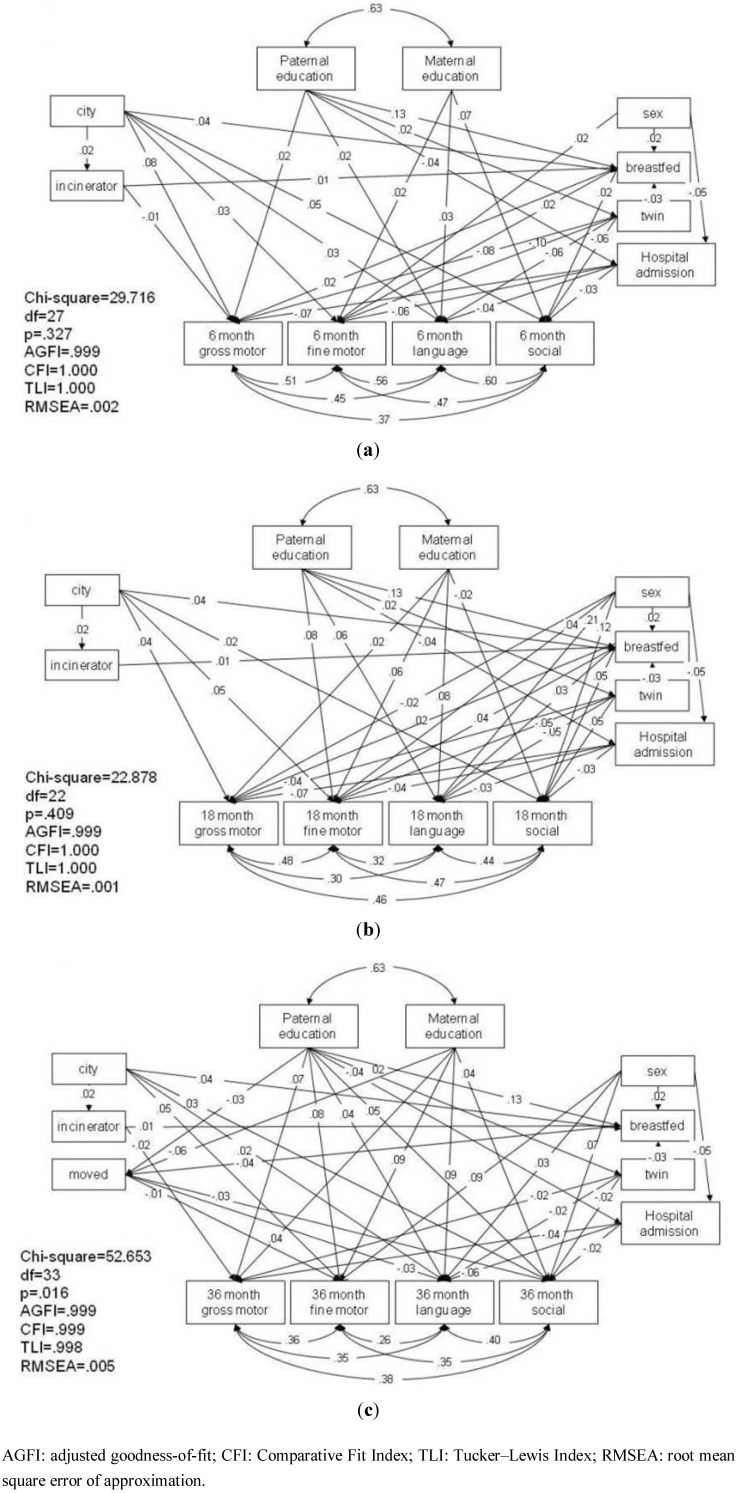
Parsimonious pathway analysis models of the relationship pathways among parental level of education, place of residence, living near an incinerator, breastfeeding, hospital admission and child development at (**a**) six months, (**b**) 18 months and (**c**) 36 months of age.

Three pathway analysis models were constructed to investigate the effect of living near an incinerator on the children’s gross motor, fine motor, language, and social development at six, 18, and 36 months, with the confounding factors controlled. The six-month and 18-month models resulted in a good fit, with p values greater than 0.05, an AGFI greater than 0.9, CFI and TLI equal to 1 and an RMSEA of less than 0.08 (Figure 2(a) and (b)), while the 36-month model showed an adequate fit, with a p value of 0.016, AGFI of 0.999, CFI and TLI close to 1 and RMSEA of 0.005 (Figure 2(c)). Living near an incinerator was associated with slower gross motor development at six and 36 months (β = –0.01, *p* = 0.061; β = –0.02, *p* = 0.007, respectively). In addition, all three models showed that living in a city was associated with a higher likelihood of living near an incinerator (β = 0.02, *p* = 0.002). However, living in a city was also associated with better child development.

Figure 2(a) shows that children who lived in the city at six months were more likely to have better gross motor (β = 0.08, *p* < 0.001), fine motor (β = 0.03, *p* < 0.001), language (β = 0.03, *p* < 0.001) and social (β = 0.05, *p* < 0.001) development than those living in rural areas. Similarly, children who lived in the city at 18 months had better gross motor (β = 0.04, *p* < 0.001), fine motor (β = 0.05, *p* < 0.001) and social (β = 0.02, *p* < 0.001) development than children living in rural areas (Figure 2(b)). Finally, Figure 2(c) shows that children who lived in the city at 36 months had better fine motor (β = 0.05, *p* < 0.001), language (β = 0.02, *p* < 0.001) and social (β = 0.03, *p* < 0.001) development than those living in rural areas. This demonstrates that children who lived in the city showed overall better development from six to 36 months.

#### 3.1.4. Pathway Analysis of Parental Concern regarding Children’s Development at 18 Months

**Figure 3:**
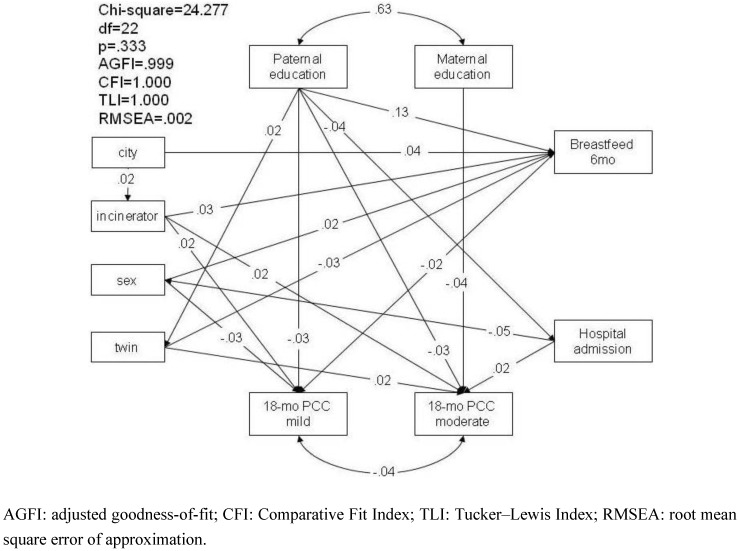
Parsimonious pathway analysis model of the relationship pathways among parental level of education, place of residence, living near an incinerator, breastfeeding, hospital admission, and parental concerns regarding child development.

The parsimonious pathway analysis for the pathway relationship between the effect of living near an incinerator and parental concern regarding child development yielded a p value of 0.333, an AGFI of 0.999, CFI and TLI of 1 and RMSEA of 0.002, which demonstrated a good fit, as shown in Figure 3. Living near an incinerator increased parental concern regarding child development for mild U/DDD (β = 0.02, *p* = 0.032) and for moderate U/DDD (β = 0.02, *p* = 0.010). In addition to direct effects, living near an incinerator had an indirect effect on U/DDD through the mediating factor of breastfeeding, which suggests that toxins can be transmitted via breastfeeding by mothers who are exposed, and children who were breastfed and living within three kilometers of an incinerator were at higher risk of showing mild U/DDD (β = 0.001).

### 3.2. Discussion

The results showed that living near an incinerator increased the risk of developmental delay in the gross motor domain at six and 36 months. In addition, more parents who lived near an incinerator reported concerns that their children had mild or moderate U/DDD at 18 months, as compared with those who did not live near an incinerator. Although children who lived in cities were, overall, perceived to be better developed than those in rural areas, there are more incinerators in cities. Therefore, children who lived in cities but within three kilometers of an incinerator showed effects on their developmental condition. In addition, although breastfeeding itself is a protective factor against U/DDD, an indirect effect was found with respect to the presence of an incinerator on U/DDD through the mediator of breastfeeding. Thus, those children who lived near an incinerator and were breastfed had an increased risk of U/DDD. However, up to the age of 36 months, no direct association was found between living near an incinerator and the parents’ perception of the level of development of the children in the cohort.

We used two different types of measure in this study: one assessed the children’s developmental milestones (TBCS-DI) and the other evaluated parental concerns (PCC). There were statistically significant differences in the perceived prevalence of mild and moderate U/DDD, using the measure of PCC, between those who lived near an incinerator and those who did not. However, only gross motor development at 36 months showed significant differences between those who lived near an incinerator and those who did not (Table 2). It should be noted that the PCC, which is a broad first-stage screening instrument, carries the risk of over-referral [24]; thus, its use might increase the apparent prevalence of disorders, with a high possibility of Type I errors. This may be especially true in the case of the PCC, because the questions relate to concerns that a parent might have regarding their child’s development. Therefore, the difference in the results between the PCC and the TBCS-DI was interesting. Nevertheless, both instruments showed that living near an incinerator increased the risk of U/DDD during child development.

Living within three kilometers of an incinerator increased the risk of children being delayed in the gross motor domain at six and 36 months of age. In addition, more parents in this group reported concerns that their children had mild or moderate U/DDD at 18 months. There was a dose effect: the presence of an incinerator had a greater effect on the group with moderate U/DDD than on the group with mild U/DDD. The present study is one of the first studies to investigate the possible effect of a local incinerator on the perceived development of young children. However, related studies show that other forms of air pollution have a detrimental effect on health. For instance, a review study from the Czech Republic found that air pollution significantly affects children’s health, especially with respect to an increase in respiratory morbidity [25]. Another Australian study of a large cohort found that exposure to traffic-related air pollution in mid to late pregnancy is associated with infants who are small for their gestational age and sex, and the proportion with optimal birth weight is below the 10th percentile [26].

As mentioned above, in addition to direct effects, living within three kilometers of an incinerator can also increase the risk of U/DDD indirectly through breastfeeding. The beneficial effects of breastfeeding on child development and child health have been demonstrated in previous studies [27,28]. Earlier studies have also proposed that the effect of breastfeeding on child development is mediated by socioeconomic status [29]. In the present study, we controlled for the level of education of the parents and found that a higher level of education of the father was associated with an increased prevalence of breastfeeding. Furthermore, the results showed that breastfeeding itself still acted as a protective effect on child development, and reduced parental concern. However, although breastfeeding is a protective factor in general, if the family lived near an incinerator, the effect of breastfeeding was inverted, and it became a risk factor for U/DDD. Previous studies have found that milk from women who live near incinerators contains traces of dioxins [14,15], which might explain the inverse effect found in the present study.

It should be noted that, to eliminate the possibility of a residual effect of six-month development on the association of living near an incinerator with 18- or 36-month development, a pathway analysis model including all three stages of development was constructed (refer to Supplemental Materials). The result of this model shows that living near an incinerator still had an association with gross motor development at 36 months. In addition, the presence of an incinerator was mediated by breastfeeding, which was indirectly associated with six-month gross motor and fine motor development and 18-month gross motor, fine motor, language and social development. This result is similar to that of the cross-sectional analysis in Figure 2, and shows that the residual effect of development at six months on later stages of development does not significantly affect the variables of interest.

In contrast to other countries, incinerators in Taiwan have been built close to cities. In Taiwan, living in a city generally implies better socioeconomic status. As a consequence, the usual association between health and exposure to waste, where disadvantaged communities in other countries suffer disproportionately from the impact of waste facilities [30], is not applicable to Taiwan. In a similar phenomenon to that described for breastfeeding, although children who lived in the city had better development at six, 18, and 36 months of age, those who lived in the city but near an incinerator had worse gross motor development at six and 36 months compared with those who lived in a city but not near an incinerator. The phenomenon of children living in cities showing better development than those living in rural areas has been found in different studies. In China, urban-rural differences have been found in the growth of breastfed babies, with those living in cities showing significantly greater weight, length and head circumference than those in rural areas [31]; urban and urbanized children have also been found to be more socially adjusted in school [32]. In the United States, increases in income were associated with great improvements in early academic skills in urban areas, but only slight improvements in rural areas [33]. As in previous studies, socioeconomic condition has been found to be an important confounding factor in child development: the level of education of the parents has been associated with the developmental condition of the children [34,35]. However, when the above factors of urbanization and socioeconomic status of parental level of education were controlled for, our study found that the environmental effect of an incinerator still had a significant impact on child development, as evaluated by different developmental domains and the level of parental concern.

A limitation of the present study is that all the information was collected from parental self-reports, including two parent-report questionnaires of child development and parental concern, and therefore potential bias may exist. However, previous studies have shown that parental reports of their concerns about their children are highly reflective of true problems [36,37]. The second limitation of this study is that the exposure to toxins was determined by whether there was an incinerator within three kilometers of the home, and therefore no information regarding the exposure of the children to specific toxins and geographic data were collected, for example by taking measurements of dietary components or the environment [38]. Thus, we are unable to suggest which toxins caused the outcome. Furthermore, there are no unified clean air standards for waste incinerators in Taiwan, because each incinerator is built and operated with assistance from technicians from the United States or the UK, thus the standards vary depending on the technical assistance provided by the respective country. However, a study of ambient air samples in Taiwan has shown that municipal solid waste incinerators are an important source of dioxin-like compounds (PCB and PBDD/Fs) [39], and similar results have also been found in Japan [40]. In addition, a 10-year follow-up study found that exposure to mercury increased as distance from a hazardous waste incinerator decreased [41]. Another limitation of this study is that we do not know how long the parents had lived near to an incinerator prior to data collection when their children were six months old, thus we are unable to determine how this may affect the children’s development. However, the factor of moving house was included in the analysis for the 36 months age group, and this factor did not have any effect on children’s development. Lastly, since a simple randomly process controlling for urbanization and fertility rate was used in our first stage of our two sampling, the sample itself is proportional to the original sample. However, no further sample weight was taken into consideration in our study, which is a limitation of our study. Although it should be noted that in Taiwan, urbanization is positively associated with high economic status and fertility rate is negatively associated with high socioeconomic status. In the British Millennium Cohort study [42], with triple the amount of total population number compared to that of Taiwan, 18,818 newborns were sampled (using a population of 63,047,162, approximately 3% selection rate). A larger sample was collected in our study (21,248 newborns, selection rate of 11.7%) because we hope that the Taiwan Birth Cohort Study (TBCS) dataset can provide us with useful information regarding predisposing and maintaining factors in rare diseases in the future.

## 4. Conclusions

Although industrialization has greatly increased the convenience of our daily lives, it is associated with certain drawbacks, which include pollution and its effects on public health. The results from our large-scale national birth cohort study show that, of the different sources of environmental pollution investigated, the presence of an incinerator was the only one that affected parent-perceived child development directly. This factor may also affect child development indirectly through the mediating factor of breastfeeding. This result lends new support to the hypothesis that environmental factors contribute to developmental delay and U/DDDs. Further follow-up of these children to investigate the long-term effects of specific toxins on their development and later diagnostic categorization is needed.

## Figures and Tables

**Supplementary Figure:**
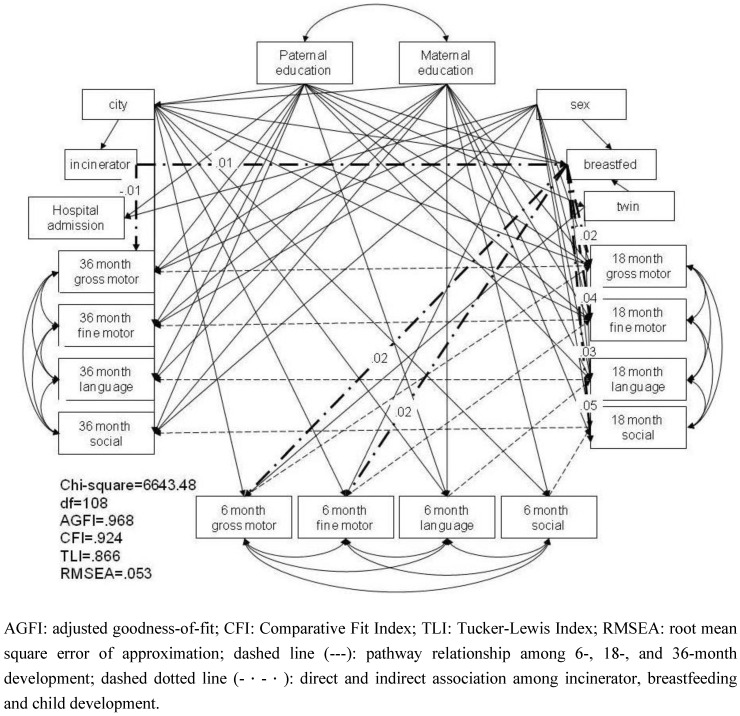
Parsimonious pathway analysis models of the relationship pathways among parental level of education, place of residence, living near an incinerator, breastfeeding, hospital admission and child development at six, 18 and 36 months of age.

## Supplemental Materials

A pathway analysis model including all three stages of development was constructed to eliminate the possibility of the residual effect of six-month development on the association of living near an incinerator with 18- or 36-month development. The model resulted in a good fit, with an AGFI greater than 0.9, CFI and TLI close to 1 and RMSEA of less than 0.08 (supplementary figure). Six-month development predicted 18- and 36-month development (represented by the dashed lines in the supplementary figure). The model is a parsimonious model, meaning that only significant pathways (*p* < 0.05) are shown. The pathway analysis showed that living near an incinerator was associated with gross motor development at 36 months (β = –0.01, *p* = 0.017): those living near an incinerator showed slower gross motor development at 36 months. In addition, living near the incinerator was associated with a higher rate of breastfeeding (β = 0.01, *p* = 0.055). Through the mediating factor of breastfeeding, the presence of an incinerator was indirectly associated with six-month gross motor (β = 0.02, *p* = 0.003) and fine motor (β = 0.02, *p* < 0.001) development and 18-month gross motor (β = 0.02, *p* = 0.011), fine motor (β = 0.04, *p* < 0.001), language (β = 0.03, *p* < 0.001) and social (β = 0.05, *p* < 0.001) development. This result shows that the residual effect of development at six months on later stages of development does not significantly affect the variables of interest.
